# A cross-country analysis of macroeconomic responses to COVID-19 pandemic using Twitter sentiments

**DOI:** 10.1371/journal.pone.0272208

**Published:** 2022-08-24

**Authors:** Zahra Movahedi Nia, Ali Ahmadi, Nicola L. Bragazzi, Woldegebriel Assefa Woldegerima, Bruce Mellado, Jianhong Wu, James Orbinski, Ali Asgary, Jude Dzevela Kong

**Affiliations:** 1 Africa-Canada Artificial Intelligence and Data Innovation Consortium (ACADIC), York University, Toronto, Canada; 2 Laboratory for Industrial and Applied Mathematics, York University, Toronto, Canada; 3 Faculty of Computer Engineering, K.N. Toosi University, Tehran, Iran; 4 Advanced Disaster, Emergency and Rapid-response Simulation (ADERSIM), York University, Toronto, Ontario, Canada; 5 School of Physics, Institute for Collider Particle Physics, University of the Witwatersrand, Johannesburg, South Africa; 6 Dahdaleh Institute for Global Health Research, York University, Toronto, Canada; University of Almería, SPAIN

## Abstract

The COVID-19 pandemic has had a devastating impact on the global economy. In this paper, we use the Phillips curve to compare and analyze the macroeconomics of three different countries with distinct income levels, namely, lower-middle (Nigeria), upper-middle (South Africa), and high (Canada) income. We aim to (1) find macroeconomic changes in the three countries during the pandemic compared to pre-pandemic time, (2) compare the countries in terms of response to the COVID-19 economic crisis, and (3) compare their expected economic reaction to the COVID-19 pandemic in the near future. An advantage to our work is that we analyze macroeconomics on a monthly basis to capture the shocks and rapid changes caused by on and off rounds of lockdowns. We use the volume and social sentiments of the Twitter data to approximate the macroeconomic statistics. We apply four different machine learning algorithms to estimate the unemployment rate of South Africa and Nigeria on monthly basis. The results show that at the beginning of the pandemic the unemployment rate increased for all the three countries. However, Canada was able to control and reduce the unemployment rate during the COVID-19 pandemic. Nonetheless, in line with the Phillips curve short-run, the inflation rate of Canada increased to a level that has never occurred in more than fifteen years. Nigeria and South Africa have not been able to control the unemployment rate and did not return to the pre-COVID-19 level. Yet, the inflation rate has increased in both countries. The inflation rate is still comparable to the pre-COVID-19 level in South Africa, but based on the Phillips curve short-run, it will increase further, if the unemployment rate decreases. Unfortunately, Nigeria is experiencing a horrible stagflation and a wild increase in both unemployment and inflation rates. This shows how vulnerable lower-middle-income countries could be to lockdowns and economic restrictions. In the near future, the main concern for all the countries is the high inflation rate. This work can potentially lead to more targeted and publicly acceptable policies based on social media content.

## 1. Introduction

More than two years has passed from the day the World Health Organization (WHO) officially declared the novel coronavirus disease known as COVID-19 a pandemic, nevertheless, despite the availability of effective vaccines, people are still getting infected and losing their lives, and countries are still placing restrictions and lockdown measurements to control the spread of the virus [1]. Since many infected people may not have any symptoms, group dining and social gathering are mostly restricted. While schools and some office jobs are converted to online teaching and remote working respectively, mass transit, hotels, restaurants, and many other businesses are still under some sort of restriction [2]. Although vaccines have successfully reduced the number of infections and deaths around the world, since the virus can spread through the vaccinated individuals, Non-Pharmaceutical Interventions (NPI) such as lockdowns still need to be applied [3, 4]. As a result of this long-term isolation and financial depression, the global economy is going through havoc and the worst is yet to come [5].

Various studies have focused on investigating the health and economic outcomes of the COVID-19 pandemic in different countries [6–11], however, understanding and contrasting macroeconomic changes across the World Bank (WB) income classes to inform better policies during the current catastrophe and prepare for future disasters has been neglected. In this paper, we aim to study how different countries from different income classes have responded to the crisis caused by COVID-19 pandemic, in terms of macroeconomic indicators such as unemployment and inflation. Based on economic development, the WB ranks and classifies countries into four groups, namely, high-, upper-middle-, lower-middle-, and low-income. Classification takes place each year on July first based on the Gross National Income (GNI) per capita of the previous year of the countries [12]. The classification was initially used for analytical purposes such as comparison and aggregation. However, today it is extended beyond that and used for cases such as loan pricing and lending decisions. GNI per capita has proven to be a good estimation for economic development and may be even more accurate than some accepted indicators of development outcomes such as Gross Domestic Product (GDP) [13]. In this work, we aim to find out how countries from different income levels have responded to COVID-19 pandemic by bringing an example from three different income levels, i.e. Nigeria for lower-middle, South Africa for upper-middle, and Canada for high income. Such analysis has been conducted for the 2009–2010 great recession [14–17]. However, to the best of our knowledge, countries have not been compared based on income and macroeconomic levels during the COVID-19 pandemic. We use the Phillips curve to study the labor market flow and price changes across income levels during the COVID-19 pandemic. Our findings on the unemployment rate using Twitter sentiments could help decision-makers determine more targeted policies for the current and future crises.

Social media has been successfully used in different fields such as behavior analysis [18], spam detection [19], electoral prediction [20], event detection [21], and economy [22]. Twitter as one of the most popular social media is widely used in micro and macroeconomics [23–25]. It has been considered an alternative way of estimating labour market metrics and statistics [26]. Census taking faces many limitations and difficulties; it is very expensive and time consuming, it requires a lot of manpower and administrative personnel and the results are usually not available in less than a month. As a result, countries carry out census only annually or seasonally. Using social media, macroeconomic metrics can be estimated and presented with less cost and effort, only using several lines of code, in real-time, and on monthly basis, rather than seasonally or annually.

Sentiment analysis is the technique of using Natural Language Processing (NLP) to identify and extract the emotion of a text. Using sentiment analysis, a text is usually classified into three different classes, i.e. positive, neutral, and negative. Finding the sentiment of the tweets related to macroeconomics could be very helpful in understanding how people feel about the labor market. Generally, lower sentiments are an indication of a worse macroeconomic situation. By combining the volume of the tweets and their sentiments we estimated the unemployment rate of Nigeria and South Africa with a higher accuracy.

In this paper, we compare three different countries from various income classes, i.e. Canada from high-income, South Africa from upper-middle income, and Nigeria from lower-middle income, in terms of macroeconomic response to the COVID-19 pandemic using the Phillips curve. Since macroeconomic factors have had shocks and rapid changes during the COVID-19 pandemic, an advantage to our work is that using Twitter data, we analyze them monthly. The inflation rate is available for all three countries monthly. Moreover, the unemployment rate is available monthly for Canada. However, the unemployment rate has been estimated quarterly for South Africa, and only twice during COVID-19 for Nigeria. There is no data available for the unemployment rate of Nigeria in 2019, but it has been reported seasonally in the years before that. To find the missing data on the unemployment rate for South Africa and Nigeria, we combine four different machine learning algorithms, namely Random Forest Regression (RFR), Support Vector Regression (SVR), eXtream Gradient Boosting (XGboost), and Auto-Regressive Integrated Moving Average with Exogeneous input (ARIMAX) to predict the unemployment rate of Nigeria and South Africa. We then use the approximated statistics to plot the Phillips curve of these countries.

Thus, in this work, we use the volume and social sentiments of tweets to estimate the unemployment rate of South Africa and Nigeria monthly. In addition, we compare the macroeconomic responses of countries from different income groups (Canada (high-income), South Africa (upper-middle income), and Nigeria (lower-middle income)) to the COVID-19 pandemic. We pursue three questions in this project:

- How have macroeconomic factors such as unemployment and inflation rates of different income classes changed during the COVID-19 pandemic compared to pre-COVID-19 time?
- How differently have the three countries, i.e. Canada (high-income), South Africa (upper-middle income), and Nigeria (lower-middle income), responded to the COVID-19 pandemic?
- What can be expected in terms of economic reaction from the three countries, each from a different income class, in the near future?

To the best of our knowledge this is the first work that compares the macroeconomic response of countries to the COVID-19 pandemic based on the WB income groups. The result of our work can provide insight to policymakers on the current macroeconomic conditions. Furthermore, it can be a lesson for future crises. By understanding the effect of lockdowns and restrictions on macroeconomic factors in different countries based on WB income level, decision-makers, small business managers, and other entities or even individuals can better prepare for future pandemics and outbreaks.

### 1.1. Literature review

Several studies have compared the performance of countries from different income levels during the COVID-19 pandemic in various aspects such as food security [27, 28] and income shocks caused by COVID-19 pandemic [29]. Studies have also compared the impact of policies measured during the pandemic across countries in different income groups [30–34]; the impact of socio-economic variables on the spread of COVID-19 [34–38]. Erokhin and Gao [27] concluded that during the COVID-19 pandemic in lower-income countries food inflation is the major crisis, while in upper-income countries food trade restrictions and currency depreciation is more frequent. In [39] it is shown that containment and closure policies resulted in less mobility, morbidity, and mortality in high and upper-middle income countries compared to low and lower-middle income countries. In [32] economic stimulus packages have been compared over different countries using beta regression and Euclidean distance.

Authors in [36–38] found a positive correlation between income inequality and the number of COVID-19 cases and deaths across all the WB income groups. In [33] it is shown that a contextualized pandemic response plan is required for Low-and-Middle-Income Countries (LMIC). In [40] demographic factors such as age, education, family size, and income for economic hardship have been studied across five different countries, Thailand, Malaysia, the UK, Italy, and Slovenia. Generally, the most vulnerable groups to the COVID-19 pandemic were people 18–24 years of age or over 65, with lower education, with children under 18, with larger family sizes, and with flexible or no income. In [29] it is shown that infant deaths have increased by 6.8% in 2020 due to COVID-19 economic contraction. Of note, none of the above studies looks at the macroeconomic responses to the COVID-19 pandemic.

Macroeconomic changes during the COVID-19 pandemic have been analyzed in some papers, but not on a cross-country-income level. Authors in [41] have used the Phillips curve in an Auto-Regressive Distributed Lag (ARDL) model to find the effect of shocks caused by lockdowns on the monetary policies, and the economy’s response to the affected policies. They concluded that to reduce the negative impacts of the COVID-19 pandemic on the economy, countries are taking less prudent monetary policies compared to before. T. W. Abate in [42] studied unemployment and inflation rate during COVID-19 in Ethiopia and concluded that because unemployment has increased as a result of lockdowns, production has decreased. This has caused a great demand for goods and therefore, inflation has also increased at the same time.

Authors in [43] argue that with a strict lockdown, the number of infections decreases, however, economic inactivity increases. In contrast, with a loose lockdown and restriction, economic inactivity decreases, but the number of infections and deaths increases. They then use the short-run Phillips curve to build a model that describes the inverse relationship between the number of COVID-19 infections and economic inactivity. Pham and Sala [44] studied the effect of inflation and unemployment rates on cross-country connectedness for the G7 countries. They found that the connectedness of inflation is high, but unemployment is low. In general, countries with higher competitiveness have a lower connectedness and do not spread their economic shocks to other countries. Furthermore, coordination in macroeconomic policies can help reduce volatility spillovers caused by shocks.

In [6] different countries such as Germany, Norway, Japan, China, Taiwan, the UK, and different cities in the USA are compared based on GDP loss and COVID-19 deaths. Countries are classified into four different groups: (1) low GDP loss and low COVID-19 deaths, (2) high GDP loss and high COVID-19 deaths, (3) high GDP loss and low COVID-19 deaths, and (4) low GDP loss and high COVID-19 deaths. They found that most of the countries and regions fall into group (1) or group (2). The reason is that due to government-mandated policies and self-protecting behaviour, countries started the pandemic with either a high or low mortality rate. Countries with a high mortality rate at the beginning of the pandemic applied stricter Non-Pharmaceutical Interventions (NPI) to reduce the number of deaths. Therefore, they suffered from both high GDP loss and high COVID-19 deaths. On the other hand, luckier countries with a low mortality rate at the beginning of the pandemic applied milder restriction policies and thus suffered less from economic loss. Therefore, they have had both low GDP loss and low COVID-19 deaths. Using epidemiological models, Phurichai [7] argued that there is a trade-off between health and mobility during an outbreak. To increase well-being, mobility must be reduced. They concluded that early implementation of NPI can effectively reduce the damages caused by a pandemic.

None of the studies mentioned above analyzed macroeconomics during the COVID-19 pandemic and across different countries incomes. Macroeconomic impacts of the global crisis have been studied before the COVID-19 pandemic based on country incomes [14–17]. However, to the best of our knowledge, this work is the first to compare the macroeconomic response of different countries to COVID-19 based on the country income groups defined by the WB.

Some papers have used social media to track economic factors such as the unemployment rate during COVID-19. In [45] three different datasets including Twitter dataset have been used to track the unemployment rate of the USA during COVID-19. The results show that the unemployment rate can be tracked using social media such as Twitter. In [26] the unemployment rate of South Africa was traced using Twitter data and Principal Component Regression (PCR) was applied to nowcast it. In this paper, we improve this method by combining four different machine learning algorithms and use the result to analyse macroeconomics during the COVID-19 pandemic compared to pre-COVID-19 years across country-income levels.

## 2. Methodology

**Fig 1:**
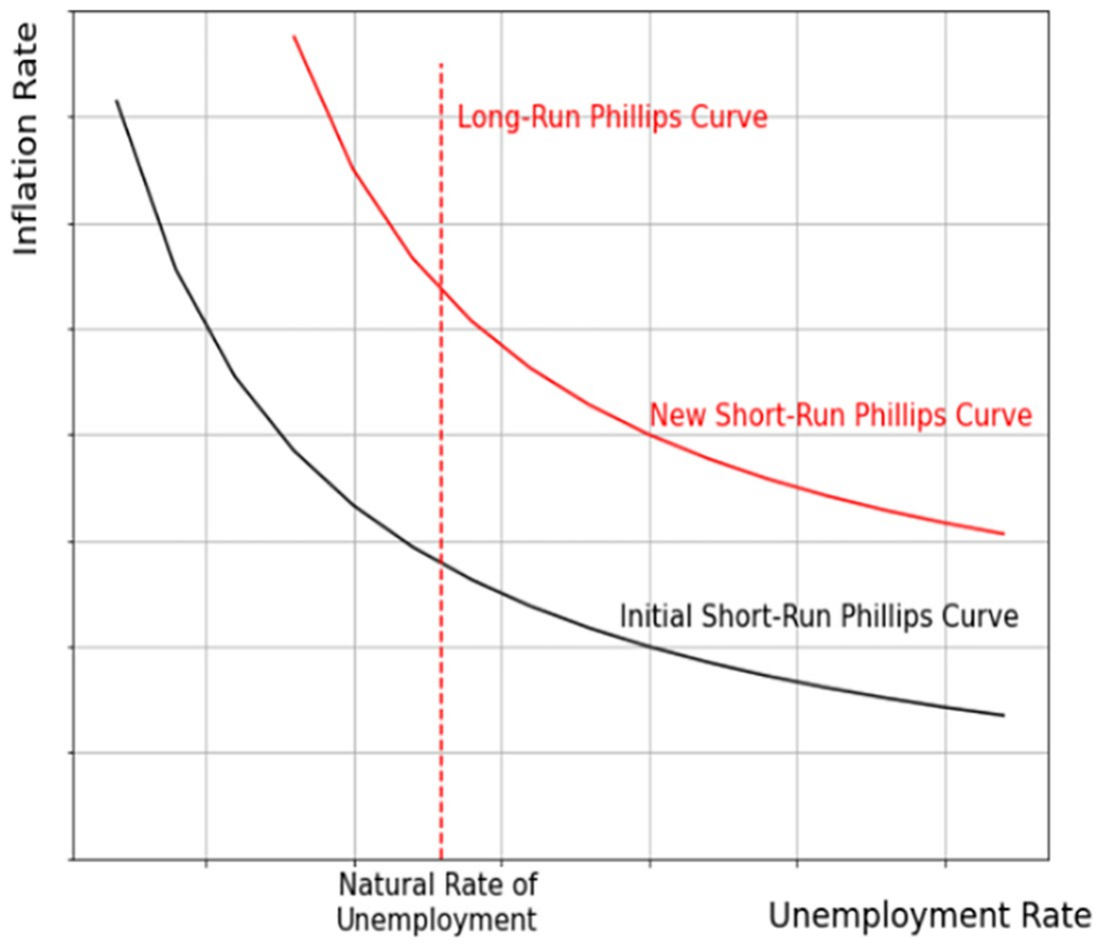
Phillips curve. The long-run and short-run Phillips curves.

The global economy has been devastated during the COVID-19 pandemic. Countries have not been affected equally by the restrictions and NPI. To study the macroeconomic changes during the COVID-19 pandemic across different country income groups, we have compared three different countries, namely, Canada from high income, South Africa from upper-middle income, and Nigeria from lower-middle income groups using the Phillips curve. Phillips curve, named after William Phillips is an empirical tool to study the inverse relationship between inflation and unemployment rates. As shown in Fig 1, the Phillips curve includes the unemployment rate on the x-axis and the inflation rate on the y-axis [46]. With a low unemployment rate, people can afford to buy more and the inflation rate increases. In contrast, during a recession, when the unemployment rate is high, people spend less money and the inflation rate decreases. However, this relation holds only in the short-run analysis [47]. In the long run, and most often during economic crises, the unemployment rate stays more or less steady regardless of the inflation rate and as displayed in Fig 1 the curve will be transformed to a new level. Therefore, as shown in Fig 1, the long-run Phillips curve is depicted as a vertical line at the natural rate of unemployment. Needless to say, curves that are closer to the zero point of the coordinates indicate a more powerful economy [48]. Therefore, it is an excellent tool for comparing countries in terms of macroeconomics. Using the Phillips curve, we can compare the macroeconomic changes during the COVID-19 pandemic with those before the COVID-19 era, and for countries from different income groups, and predict the near future.

Due to on and off rounds of lockdowns, during the COVID-19 pandemic, macroeconomic indicators, especially unemployment rate have had rapid changes and fluctuations. Therefore, we aim to study the Phillips curve of the three countries from different income groups on monthly basis.

### 2.1. Unemployment and inflation rates for Canada

We obtained monthly data for the unemployment and inflation rates for Canada from [49, 50].

### 2.2. Unemployment and inflation rates for Nigeria and South Africa

Monthly data for the inflation rates for Nigeria and South Africa were obtained from [51] and [52], respectively. Since the unemployment rate of Nigeria and South Africa are not available monthly, we use social media to find the missing information. Social media is increasingly becoming popular. People share their thoughts, experiences, and concerns on social media. Therefore, it is a great source of information that can help retrieve data and derive different indicators such as the unemployment rate. We use a method similar to the one used in [26] to find the unemployment rates of Nigeria and South Africa. According to the International Labor Organization (ILO) unemployment rate can be estimated using Google Mobility Index (GMI) for countries with limited data [53]. GMI is an indicator that shows how many people have moved around six different locations, namely, retail and recreation, groceries and pharmacies, parks, transit stations, workplaces, and residential [54]. It has been temporarily available during the COVID-19 pandemic to help different sectors, especially health workers in making decisions. According to ILO and other references [6, 7], the unemployment rate of countries with limited data can be estimated using GMI [53]. However, in this work, we use GMI to only gather our dataset, not to estimate the unemployment rate. We use certain keywords related to the unemployment rate to search for geotagged tweets from Nigeria and South Africa. We used the same set of keywords in [26], i.e. unemployed, employed, and different forms of retrenching to find a dataset for South Africa. For Nigeria, we used the keywords employed, unemployed, and lost * job(s), where * indicates a wildcard, i.e. lost his job, lost her job, lost my job, and lost their jobs to gather a dataset.

The number of tweets with the mentioned keywords and the sum of sentiment scores of the tweets were respectively, positively and negatively correlated with GMI during the COVID-19 pandemic and quarterly unemployment rate before COVID-19 for Nigeria and South Africa. We used the volume of the tweets and their sentiment scores to predict the unemployment rate of Nigeria and South Africa with different machine learning algorithms.

Sentiment analysis is a text classification technique that classifies the sentiment of a text into negative, neutral, or positive sentiment. A sentiment score is a number between -1 and 1. Positive, neutral, and negative sentiments have a number close to 1, 0, and -1, respectively. Unlike in [26] where the authors used a pre-trained model of Bidirectional encoder Representation of Transformers (BERT) to perform sentiment analysis, we trained our model for sentiment analysis using BERT and used it to find unemployment rates of Nigeria and South Africa to compare the macroeconomic response of countries from different income levels during COVID-19 pandemic. There are two BERT models available for fine-tuning, BERT-large and BERT-base. We used BERT-base to fine-tune the model to our dataset. While the pretrained model used in [26] had 69% accuracy, our model had 76% accuracy in our dataset. Detailed information on our sentiment analysis model using BERT is available in S1 Appendix.

We applied different machine learning algorithms to the volume of the tweets and their sentiments to estimate the unemployment rate of Nigeria and South Africa, and depict their Phillips curves to analyze the macroeconomic changes. We used the average results of the four machine learning algorithms, i.e. RFR, SVR, XGBoost, and ARIMAX to predict the unemployment rate of Nigeria and South Africa. From the beginning of 2017 till now, the census unemployment rate is available and known for 9 and 19 months for Nigeria and South Africa, respectively. Starting from the second know month, we used the previous known month(s) to train the machine learning model and predict the unemployment rate for the coming months. Our algorithm for predicting the unemployment rate of South Africa and Nigeria using census measures is available in S1 Appendix (Fig A-5 in S1 Appendix).

We use the data on the unemployment and inflation rates to depict the Phillips curve for Canada, South Africa, and Nigeria, and compare and analyze the unemployment and inflation rate responses of these countries before and during the COVID-19 pandemic.

## 3. Results and discussion

### 3.1. Data gathering and sentiment analysis

**Fig 2:**
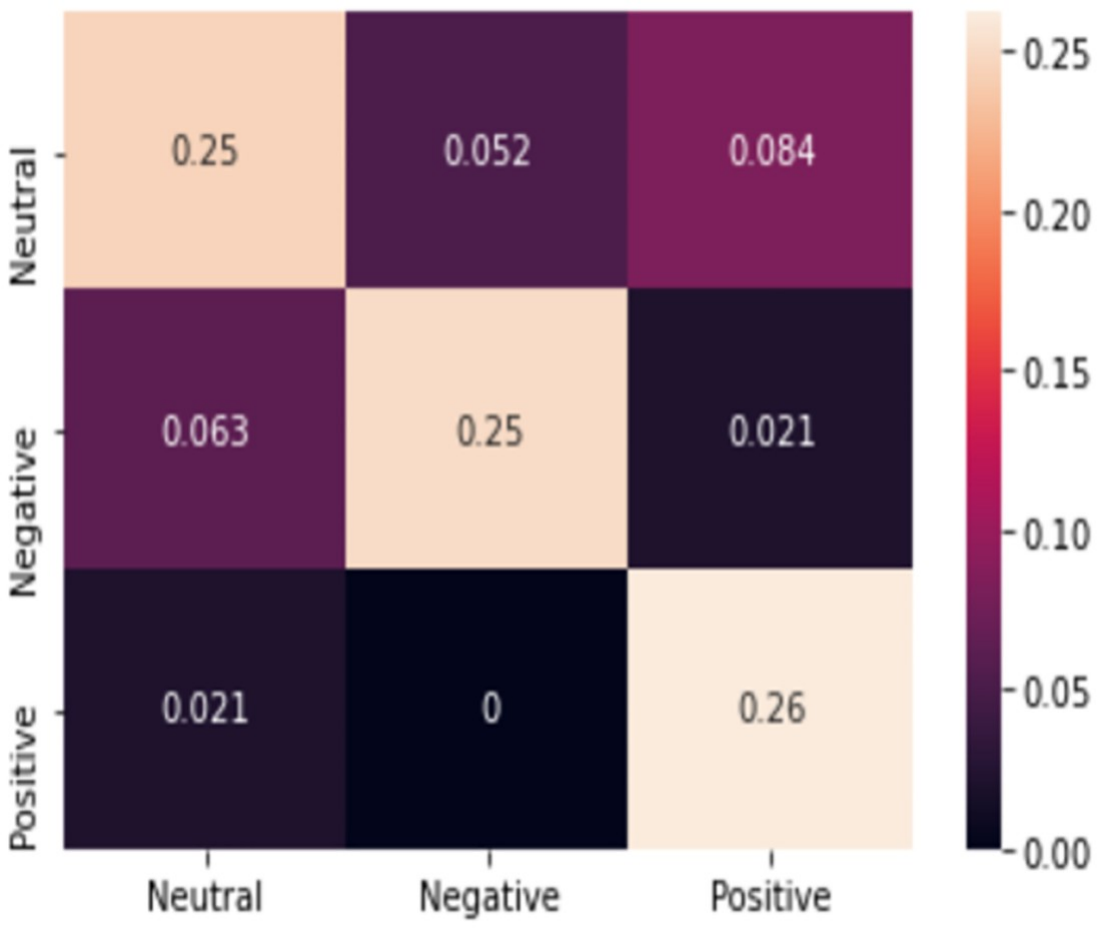
Confusion matrix. The confusion matrix of the sentiment analysis model.

The number of tweets with all the keywords had a relatively strong correlation with the unemployment rate and GMI, respectively before and during COVID-19 for Nigeria and South Africa. Moreover, the total dataset had a strong correlation with the unemployment rate. Therefore, we used the volume of the tweets to predict the unemployment rates of Nigeria and South Africa. Next, we fine-tuned a sentiment analysis model using BERT and found 76% accuracy on our dataset. Fig 2 shows the confusion matrix of our model and Table 1 shows the different metrics of it.

**Table 1:** Machine learning metrics. Different metrics of the sentiment analysis model.

	Recall	Precision	F1-Score
**Neutral**	0.7	0.64	0.67
**Negative**	0.61	0.75	0.67
**Positive**	0.54	0.93	0.69

From the confusion matrix in Fig 2, we can see that the probability of our predictions not being accurate is pretty low. In addition, according to Table 1, our model has a high recall and precision, meaning that it is able to find each class (precision) and every one (recall) pretty well. Moreover, a high F1-score indicates that both recall and precision are high for all of the classes.

The sum of sentiment scores of the tweets had a negative correlation with the unemployment rate before and during COVID-19 pandemic for both South Africa and Nigeria. This was expected because as the unemployment rate increases, people become dissatisfied and sentiments decrease, and vice versa [26]. Table 2 shows the correlation between the sentiments and unemployment rate and our dataset for the period before and during COVID-19 pandemic for Nigeria and South Africa. In addition, Table 2 compares our trained BERT model with the pretrained BERT model used in [26]. The results show that the sentiment scores of our model have a higher correlation with the unemployment rate of South Africa before and during the COVID-19 pandemic. Detailed information can be found in S1 Appendix.

**Table 2:** Correlation checking. Correlation of the sentiment scores with unemployment rate and the dataset for Nigeria and South Africa.

	Unemployment rate	employed	unemployed	retrench	lost * job	Total dataset
Sentiments before COVID-19	**-0.81**	-0.98	-0.99	-	-0.67	-0.97
Sentiments during COVID-19	**-0.72**	-0.95	-0.93	-	-0.79	-0.98
Sentiments with our model before COVID-19	**-0.94**	-0.97	-0.96	-0.9	-	-0.99
Sentiments with model in [26] before COVID-19	**-0.71**	-0.9	-0.92	-0.56	-	-0.93
Sentiments with our model during COVID-19	**-0.81**	-0.94	-0.92	-0.71	-	-0.98
Sentiments with model in [26] during COVID-19	**-0.67**	-0.98	-0.98	-0.86	-	-0.99

Unfortunately, GMI is not available for pre-COVID-19 times. Therefore, the unemployment rate cannot be estimated using that for the period before COVID-19. Moreover, it takes several days for GMI to get updated. However, Twitter data can be accessed in real-time. Therefore, social media is alternatively used to find the missing data on unemployment rate for South Africa and Nigeria.

### 3.2. Estimating unemployment rate

We applied four different machine learning algorithms, namely, RFR, SVR, XGBoost, and ARIMAX on the datasets for Nigeria and South Africa to predict the unemployment rates of the countries. With RFR, we searched for around ten different decision trees to find the best model, since increasing the number of trees to more than ten did not improved the accuracy. With SVR, we used Radius-Basis Function (RBF) as the kernel. Moreover, the best order that we found for ARIMAX was (1, 0, 1). We found that combining the four algorithms by averaging their results has the highest performance. Table 3 shows the performance of the different algorithms. We have compared the models in terms of Mean Average Percentage Error (MAPE), Root Mean Square Error (RMSE), and R^2^-score [55].

**Table 3:** Method evaluation. Accuracy of different machine learning algorithms used for predicting the unemployment rate.

	Nigeria			South Africa		
	MAPE	RMSE	*R*^*2*^-Score	MAPE	RMSE	*R*^*2*^-Score
RFR	0.21081	5.82115	-	0.07682	8.53945	-
SVR	0.20847	5.76952	-	0.08212	8.60633	-
XGBoost	0.23170	6.05365	-	0.09956	8.80322	-
ARIMAX	0.26649	7.02462	-	0.10152	8.82080	-
**Average**	**0.06364**	**1.57671**	**0.91382**	**0.02984**	**2.01470**	**0.5753**

As can be seen in Table 3, averaging over all the machine learning methods reduces MAPE and RMSE by up to 76, 77 percent for Nigeria and 71, 77 percent for South Africa, and significantly increases R^2^-score. We used the estimated values to draw the Phillips curve for Nigeria and South Africa.

### 3.3. Analysis of macroeconomic responses to COVID-19 pandemic

**Fig 3:**
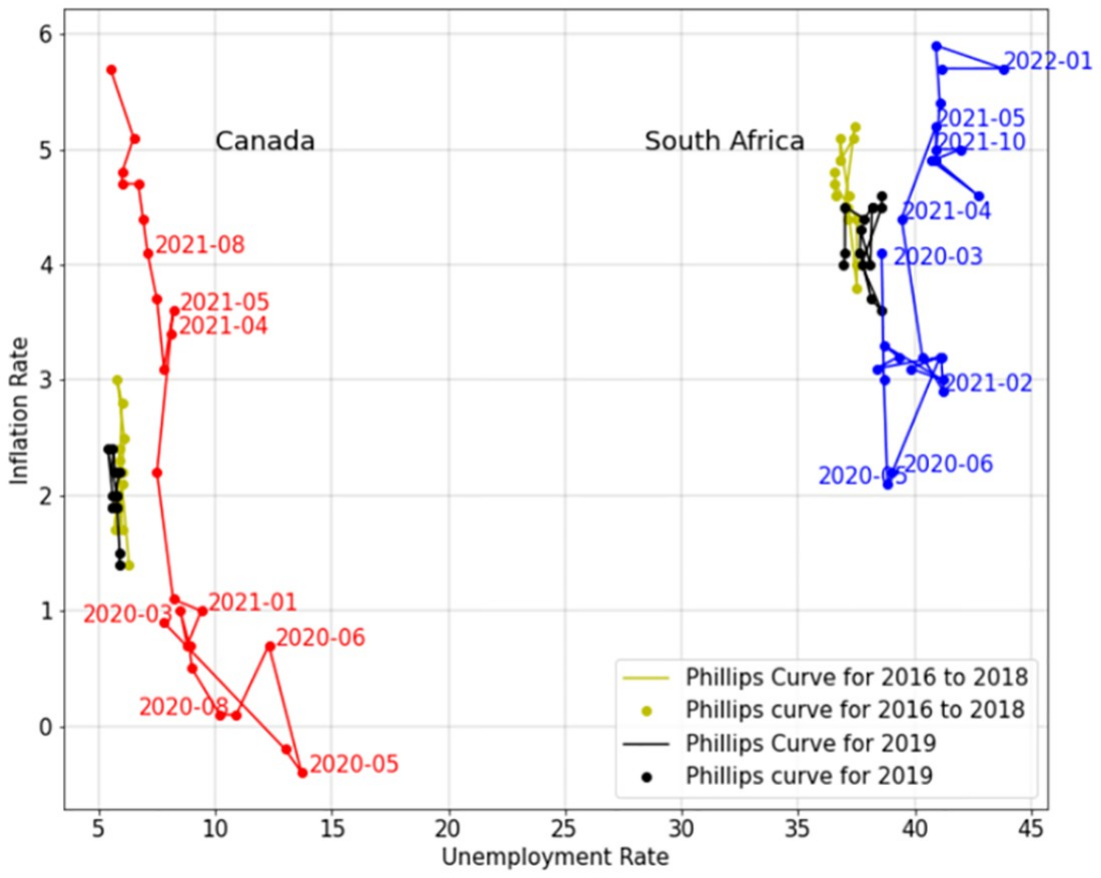
Phillips curve. Phillips curve of Canada and South Africa.

**Fig 4:**
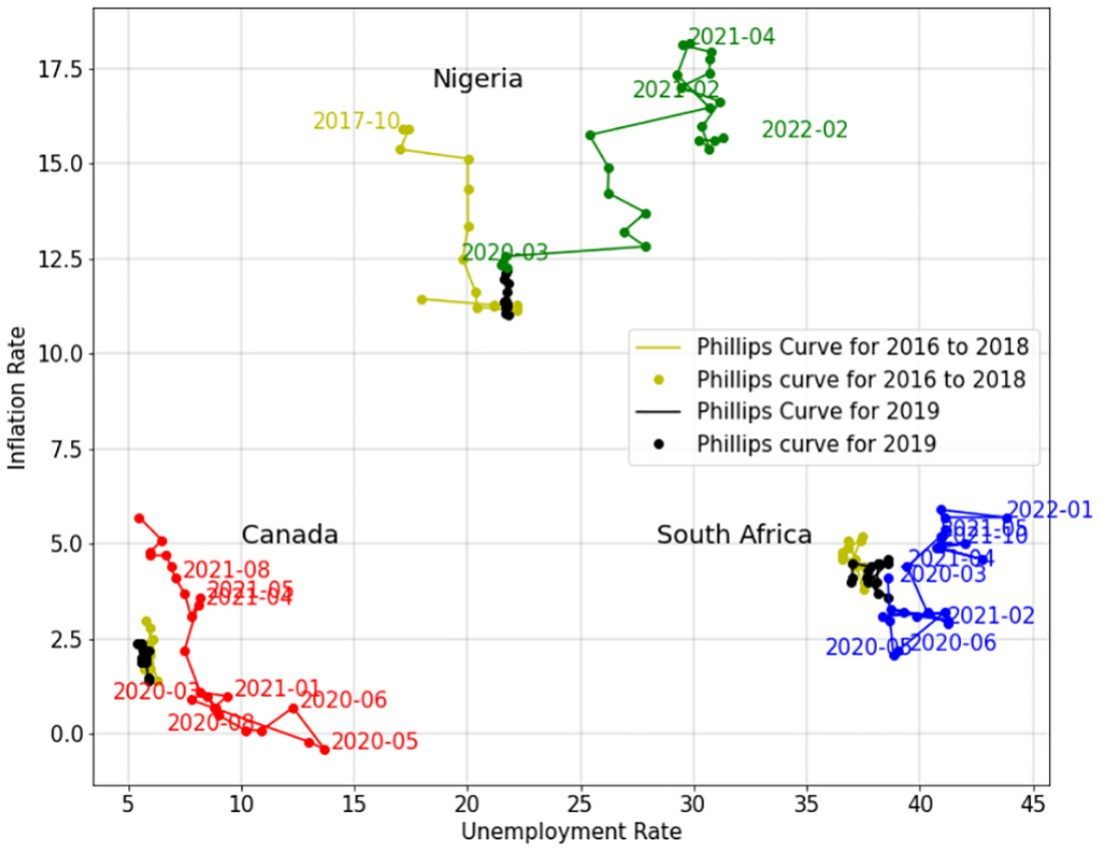
Phillips curve. Phillips curve of Canada, South Africa, and Nigeria.

Fig 3 shows the Phillips curve for Canada and South Africa and Fig 4 shows that for Canada, South Africa, and Nigeria.

In Figs 3 and 4, The red, blue, and green lines are the Phillips curves for Canada, South Africa, and Nigeria during the COVID-19 pandemic, respectively. The black and yellow lines are the Phillips curves for years 2019 and 2017–2018 for the given country. Since the diagram of Canada is closer to the zero point of the coordinates, it is clear that Canada has a better economy compared to South Africa and Nigeria. Moreover, before COVID-19, the unemployment rate of Nigeria was lower than South Africa and the inflation rate of South Africa was lower than Nigeria. However, during COVID-19 pandemic, unemployment and inflation rates of Nigeria has increased more than Canada and South Africa.

As shown in Figs 3 and 4, in all the three countries, the unemployment rate has increased during 2020 compared to the previous years. For Canada and South Africa from the beginning of the pandemic until May 2020, with the unemployment rate rising, inflation rate decreased. This is in line with the Phillips curve short run. Since May 2020, Canada has been able to control and reduce the unemployment rate, but South Africa and Nigeria are still struggling with the high unemployment rate caused by the pandemic. With employment rate reducing for Canada, the inflation rate has increased. This again is in agreement with the Phillips curve short run. However, the inflation rate of Canada, especially after March 2021 has increased to its highest in more than fifteen years. Other than reduction of supplements during COVID-19 pandemic, the policies that Canada informed during COVID-19 pandemic by providing Canada implemented such as providing the Canada Emergency Response Benefit (CERB) and Employment Insurance (EI) to the employees who lost their jobs may have caused this high inflation rate.

Since May 2020, unemployment rate in South Africa has not decreased to pre-COVID-19 level, but the inflation rate has increased. Although inflation rate for South Africa is still comparable to pre-COVID-19 years, according to Phillips short run curve, it will increase as the unemployment rate decreases, and it may get higher than the years before COVID-19 pandemic.

Unemployment and inflation rates in Nigeria have increased fiercely compared to Canada and South Africa during the pandemic, and the country is experiencing a horrible stagflation. This shows how vulnerable lower-middle income countries could be to lockdowns, restrictions, and economic limitations.

As stated in previous works [14–17], in 2008–2009 global economic crisis, higher-income countries suffered more from the global recession. However, the economic burden of COVID-19 crisis is heavier on lower income countries. The reason is that in the 2008–2009 crisis, the recession was caused by the impairment of the communication net between countries, especially with the USA. However, the macroeconomic complexities of the COVID-19 pandemic are caused by lockdowns and economic inactivity. As a result, countries with less economic asset bear heavier setbacks.

Overall, in the near future, unemployment may not increase that much for different countries anymore. However, all the countries, no matter which income level they belong to, will most probably experience a rise in inflation rate due to supplement shortages caused by the pandemic.

## 4. Conclusion and future work

During the COVID-19 pandemic macroeconomic factors, including the labor market metrics and especially the unemployment and the labor force participation rates have changed rapidly. This has affected each country’s economic, financial, and industrial sector; and socio-demographic group. Some jobs have been destroyed by the coronavirus causing the inflation to appear more complex, nonlinear, and subject of academic debate. A wide array of inflationary and deflationary factors such as supply chain disruption, decrease in goods production, and demand fluctuations have simultaneously made the effects of the COVID-19 pandemic unanticipatable. This has caused an increase in inflation rate across different country income groups around the globe, although unemployment rate has become stable to some extent in many countries. Therefore, the Phillips curve of many countries has acted like the long-run, and as a result, risen to a new level. Coming back to the previous level will be very difficult if not impossible. Any decrease in unemployment rate would cause an increase in the inflation rate and vice versa.

In this work, we compared three countries from different income levels, namely, Canada (high income), South Africa (upper-middle income), and Nigeria (lower-middle income) with each other using the Phillips curve. Inflation rate is available on a monthly basis for all the three countries. Moreover, the unemployment rate has been reported on a monthly basis for Canada. However, unemployment rate is not available on a monthly basis for South Africa and Nigeria. During COVID-19 pandemic, macroeconomic factors and especially unemployment rate have had rapid fluctuations. Census measurements are not able to captures these changes. Moreover, it takes months for the results to be reported. Due to the difficulties involved in conducting census, COVID-19 caused come countries (such as Nigeria) to go for two years without conducting a census. Using social media, we have estimated the unemployment rates of Nigeria and South Africa pretty well, with less cost and effort, and in real-time. This can be highly beneficial for macroeconomic analysis and informing policies. Using the volume of the tweets and their sentiments, we found the Phillips curve for the three countries.

The results show that in the first two months of the pandemic, the unemployment rate increased in all the three countries. With the unemployment rate increasing, inflation rate decreased in Canada and South Africa which is in-line with the Phillips curve short run. After the first two months, Canada was able to control the unemployment rate, however, South Africa and Nigeria are still struggling with that. By decreasing the unemployment rate, the inflation rate of Canada has significantly increased. Canada has not experienced such a high inflation rate in more than fifteen years. The inflation rate of South Africa is still comparable to the years before COVID-19 pandemic. Nonetheless, based on the Phillips curve short run, if South Africa implements policies to reduce the unemployment rate, it may face a further increase in the inflation rate. Moreover, unemployment and inflation rates have badly increased during the pandemic for Nigeria causing a wild stagflation. This indicates how vulnerable lower-middle income countries are to lockdowns and economic limitations, bearing a greater loss during the COVID-19 pandemic. All the three countries will most likely be dealing with a high inflation rate, in the near future. Although unemployment rate is not increasing as fast as the early pandemic times, inflation rate is significantly increasing in all the three countries, resulting in the Phillips curve acting as its long run. Coming back from this complexity will be very difficult, especially for middle-income countries. In due course, according to the Phillips curve, the COVID-19 crisis has affected all income country groups, however, the burden is heavier on lower income classes.

Macroeconomic indicators can be effectively combined with unconventional data analysis, including tweets’ sentiment analysis to monitor the effects of the COVID-19 pandemic on the global and local economy in real-time. Decision- and policy-makers can exploit such information to inform their decision-making processes in a data-driven and evidence-based fashion. Data from Twitter as well as other social media and social networks can indeed be utilized to better understand concerns and worries concerning the macroeconomic situations at the local level. This can potentially lead to more targeted and publicly acceptable policies based on social media content. However, despite their promising potentials, these kinds of data are relatively underlooked in the existing scholarly literature. Saleh et al. [56] as well as Oyebode and coworkers [57] and Li and colleagues [58], confirming our findings, reported that a significant portion of tweets and posts on social networkers expressed fear of the societal implications of the COVID-19 pandemic, conveying worries and concerns for the loss of income, unemployment, inflation, and financial burden/hardship.

As a contribution to the future of our work, we can use other curves such as Beveridge curve to compare countries from different income levels during COVID-19 pandemic. This could be extremely important because many jobs have been totally destroyed during the pandemic. For this purpose, social media can be used to estimate job vacancy and unemployment rates.

## Supporting information

S1 Appendix It explains how Twitter data was gathered and analyzed. (PDF)

S1 File Nigeria.csv. (TXT)

S2 File SouthAfrica.csv. (TXT)

## References

[1] Kim J H, Marks F, Clemens J D, Looking beyond COVID-19 vaccine phase 3 trials, *nature medicine*, 2021;27. doi: 10.1038/s41591-021-01230-y 33469205

[2] Musselwhite C, Avineri E, Susilo Y, Restrictions on mobility due to the coronavirus Covid19: Threats and opportunities for transport and health, Elsevier, 2021;20. doi: 10.1016/j.jth.2021.101042 33717983 PMC7936547

[3] Betti M, Bragazzi N, Heffernan L, Kong J, Raad A, Integrated vaccination and non-pharmaceutical interventions based strategies in Ontario, Canada, as a case study: a mathematical modeling study, J. of The Royal Society Interface. 2021;18(180). doi: 10.1098/rsif.2021.0009 34255985 PMC8277469

[4] Moore S, Hill E M, Tildesley M J, Dyson L, Keeling M J, Vaccination and non-pharmaceutical interventions for COVID-19: a mathematical modelling study, Elsevier, Infectious Diseases, 2021;21(6). doi: 10.1016/S1473-3099(21)00143-2 33743847 PMC7972312

[5] Bailey N W, West D, Are the COVID19 restrictions really worth the cost? A comparison of estimated mortality in Australia from COVID19 and economic recession, arXiv:2005.03491, Physics and Society, 2020.

[6] Fernández-Villaverde J, Jones C I, Macroeconomic outcomes and COVID-19: a progress report (No. w28004). National Bureau of Economic Research, 2020.

[7] Rungcharoenkitkul P, Macroeconomic effects of COVID‐19: A mid‐term review. Pacific Economic Review, 2021;26(4).

[8] Fan L, Yu H, Yin Z, Stigmatization in social media: Documenting and analyzing hate speech for COVID‐19 on Twitter. Proceedings of the Association for Information Science and Technology, 2020;57(1), e313. doi: 10.1002/pra2.313 33173820 PMC7645876

[9] Díaz F, Henríquez P A, Social sentiment segregation: Evidence from Twitter and Google Trends in Chile during the COVID-19 dynamic quarantine strategy. PloS one, 2021;16(7), e0254638. doi: 10.1371/journal.pone.0254638 34255804 PMC8277056

[10] Rowe F, Mahony M, Graells-Garrido E, Rango M, Sievers N, Using Twitter to track immigration sentiment during early stages of the COVID-19 pandemic. Data & Policy, 2021;3.

[11] Vydra S, Kantorowicz J, Tracing Policy-relevant Information in Social Media: The Case of Twitter before and during the COVID-19 Crisis. Statistics, Politics and Policy, 2021.

[12] The World Bank, World bank country and lending groups, country classification, 2021 [Cited Nov 2021]. https://datahelpdesk.worldbank.org/knowledgebase/articles/906519-world-bank-country-and-lending-groups

[13] Fantom N, Serajuddin U, The world bank’s classification of countries by income, Policy Research working paper, no. WPS 7528 Washington, D.C., World Bank Group, 2016;1(1).

[14] Aizenman J, Pasricha G K, Determinants of financial stress and recovery during the great recession, *NBER*, (2010).

[15] Lane PR, Milesi-Ferretti G M, The Cross-Country Incidence of the Global Crisis, IMF Economic Review, 2010. doi: 10.1057/imfer.2010.12

[16] Rose A K, Speigel M M, Cross-Country Causes and Consequences of the 2008 Crisis: Early Warning, NBER, (2009).

[17] Rose, A K, Speigel M M, Cross-Country Causes and Consequences of the 2008 Crisis: An Update, NBER, 2010.

[18] Arora A, Bansal S, Kandpal C, Aswani R, Dwivedi Y, Measuring social media influencer index- insights from Facebook, Twitter and Instagram, Elsevier, J. of Retailing and Consumer Services, 2019;49. doi: 10.1016/j.jretconser.2019.03.012

[19] Aswani R, Kar A K, Ilavarasan PV, Detection of spammers in twitter marketing, A hybrid approach using social media analytics and bio inspired computing, Springer, Information Systems Frontiers, 2018. doi: 10.1007/s10796-017-9805-8

[20] Singh P, Kumar K, Kahlon K S, Sawhney RS, Can Tweets predict election results? Insights from Twitter analytics, Springer, Advanced Informatics for Computing Research, 2019;1075. doi: 10.1007/978-981-15-0108-1_26

[21] Zhang Y, Shirakawa M, Hara T, A general method for event detection on social media, *Springer*, *Advances in Databases and Information Systems*, 2021;12843. doi: 10.1007/978-3-030-82472-3_5

[22] Ray P, Chakrabarti A, Ganguli B, Das PK, Demonetization and its aftermath: an analysis based on twitter sentiments, Springer, Sādhanā, 2018;43.

[23] Shahzad Q, Nooraini Y, Farzana KA, Ramsha A, Sentiment analysis of impact of technology on employment from text on Twitter, *iJIM*, *International J*. *of Interactive Mobile Technologies*, 2020;14(7). doi: 10.3991/ijim.v14i07.10600

[24] Ebrahimi P, Salamzadeh A, Gholampour A, Fekete-Farkas M, Social networks marketing and Hungarian online consumer purchase behavior: the microeconomics strategic view based on IPMA matrix, Academy of Strategic Management Journal, 2021;20(4).

[25] Ryu P-M, Predicting the unemployment rate using social media analysis, J. of Information Processing Systems, 2018;14(4).

[26] Nia Z M, Asgary A, Bragazzi N, Melado B, Orbinski J, Wu J, et al., Tracing unemployment rate of South Africa during the COVID-19 pandemic using Twitter data, JMIR Preprints, 2021. 10.3389/fpubh.2022.952363 PMC9757491 36530702

[27] Erokhin V, Gao T, Impacts of COVID-19 on trade and economic aspects of food security: evidence from 45 developing countries, *Int J Environ Res Public Health*, 2020;17(16). doi: 10.3390/ijerph17165775 32785155 PMC7459461

[28] Josephson A, Kilic T, Michler J D, Socioeconomic impacts of COVID-19 in low-income countries, Nat Hum Behav, 2021. doi: 10.1038/s41562-021-01096-7 33785897

[29] Shapira G, de Walque D, Friedman J, How many infants may died in low-income and middle-income countries in 2020 due to the economic contraction accompanying the COVID-19 pandemic? Mortality projections based on forecasted declines in economic growth, BMJ Open, 2021. doi: 10.1136/bmjopen-2021-050551 34429282 PMC8413467

[30] Shafiullah M, Khalid U, Chaudhry S M, Do stock markets play a role in determining COVID-19 economic stimulus? A cross-country analysis, *The World Economy*, 2021. doi: 10.1111/twec.13130 34230757 PMC8250644

[31] Thorpe J, Viney K, Hensing G, Lonnroth K, Income security during periods of ill health: a scoping review of policies, practice and coverage in low-income and middle-income countries, BMJ Globe Health, 2020. doi: 10.1136/bmjgh-2020-002425 32540963 PMC7299014

[32] Siddik N A, Economic stimulus for COVID-19 pandemic and its determinants: evidence from cross-country analysis, Helliyon, 2020. doi: 10.1016/j.heliyon.2020.e05634 (2020). 33319096 PMC7724168

[33] Grid COVID-19 Study Group, Combating the COVID-19 pandemic in a resource-constrained setting: insights from initial response in India, *BMJ Glob Health*, 2020. doi: 10.1136/bmjgh-2020-003416 33187963 PMC7668115

[34] Duhon J, Bragazzi N, Kong J D, The impact of non-pharmaceutical interventions, demographic, social, and climatic factors on the initial growth rate of COVID-19: A cross-country study, Elsevier, Science of the Total Environment, 2021. doi: 10.1016/j.scitotenv.2020.144325 33338848 PMC7728414

[35] Paprottka F J, Rolfes S B, Richter D F, Kaye K O, COVID-19 pandemic: evaluation of socio-economic impact on aesthetic plastic surgery providers, Aesthetic Plat Surg, 2021. doi: 10.1007/s00266-021-02130-9 33830307 PMC8029606

[36] Wildman J, COVID-19 and income inequality in OECD countries, *Eur J Health Econ*., 2021;22 (3). doi: 10.1007/s10198-021-01266-4 33590424 PMC7883879

[37] Elgar F J, Stefaniak A, Wohl M J A, The trouble with trust: time-series analysis of social capital, income inequality, and COVID-19 deaths in 84 countries, Elsevier, Social Science & Medicine, 2021;263. doi: 10.1016/j.socscimed.2020.113365 32981770 PMC7492158

[38] Kong J D, Tekwa E. W., Gignoux-Wolfsohn S. A., Social, economic, and environmental factors influencing the basic reproduction number of COVID-19 across countries, Plos One, 2021;16(6). doi: 10.1371/journal.pone.0252373 34106993 PMC8189449

[39] Pincombe M, Reese V, Dolan C B, The effectiveness of national-level containment and closure policies across income levels during the COVID-19 pandemic: an analysis of 113 countries, 2021. 10.1093/heapol/czab054 PMC8135717 33942081

[40] Osterrieder A, Cuman G, Pan-Ngum W, Cheah PK, Cheah P-K, Peerawaranun P, et al. Economic and social impacts of COVID-19 and public health measures: results from an anonymous online survey in Thailand, Malaysia, the UK, Italy and Slovenia, BMJ Open, 2021. doi: 10.1136/bmjopen-2020-046863 34285007 PMC8295020

[41] Ma Y, Chen Z, Mahmood MT, Shahab S, The monetary policy during shocks: an analysis of large Asian economies’ response to COVID-19, Taylor & Francis, Economic Research- -Ekonomska Istraživanja, 2021. doi: 10.1080/1331677X.2021.1926304

[42] Abate TW, The effect of COVID-19 on unemployment and price changes in ethiopia: evidence from woldia town “does the downward sloping Philips curve hold true”, *Economics*, 2020:9 (4). doi: 10.11648/j.eco.20200904.11

[43] Fukao M, Shioji E, Is there a trade-off between COVID-19 control and economic activity? implications from the Phillips curve debate, Wiley, Asian Economic Policy Review, 2021. doi: 10.1111/aepr.12361

[44] Pham BT, Sala H, Cross-country connectedness in inflation and unemployment: measurement and macroeconomic consequences, Springer, Empirical Economics, 2021. doi: 10.1007/s00181-021-02052-0 33897094 PMC8056377

[45] Rizio D, Suryavanshi T, Yahya M, Garg V, Can we use Twitter to track COVID-caused unemployment in the USA?, 2021, Data Science Report.

[46] Forder J, Textbooks on the Phillips curve, History of Political Economy, 2015;47(2). doi: 10.1215/00182702-2884309

[47] Graham L, Snower D J, The return of the long-run Phillips curve, SSRN, 2003.

[48] McLeay M, Tenreyro S, Optimal inflation and the identification of the Phillips curve, *NBER*, 2019;34. doi: 10.1086/707181

[49] Statistics Canada, The daily and key data tables, Labour force survey, Dec. 2021, [Cited: Dec. 2021]. https://www.statcan.gc.ca/en/bcp/daily-key-data-tables

[50] Statistics Canada, The daily and key data tables, Consumer Price Index, Dec. 2021, [Cited: Dec. 2021]. https://www.statcan.gc.ca/en/bcp/daily-key-data-tables

[51] Nigerian Stat, National Bureau of Statistics, E-Library, CPI and Inflation Report November 2021, 2021, [Cited: Dec 2021]. https://nigerianstat.gov.ng/elibrary?queries=price

[52] Statistics South Africa, Inflation, 2021, [Cited: Dec 2021]. http://www.statssa.gov.za/?cat=33

[53] International Labour Organization, ILO Monitor: COVID-19 and the world of work. Seventh edition Updated estimates and analysis, ILO, 2021, https://www.ilo.org/wcmsp5/groups/public/—dgreports/—dcomm/documents/briefingnote/wcms_767028.pdf

[54] Google, See how your community is moving around differently due to COVID-19, COVID-19 Community Mobility Reports, 2020, [Cited March 2022], https://www.google.com/covid19/mobility/

[55] Chicco D, Warrens MJ, Jurman G, The coefficient of determination R-squared is more informative than SMAPE, MAE, MAPE, MSE and RMSE in regression analysis evaluation, PeerJ Computer Science. 2021;7(e623). doi: 10.7717/peerj-cs.623 34307865 PMC8279135

[56] Saleh SN, Lehmann CU, McDonald SA, Basit MA, Medford RJ, Understanding public perception of coronavirus disease 2019 (COVID-19) social distancing on Twitter, *Cambridge Core*, *ICHE*, 2020;42(2). doi: 10.1017/ice.2020.406 32758315 PMC7450231

[57] Oyebode O, Ndulue C, Adib A, Mulchandani D, Suruliraj B, Orji FA, et al. Health, psychosocial, and social issues emanating from the COVID-19 pandemic based on social media comments: text mining and thematic analysis approach, JMIR Med Inform, 2021;9 (4). doi: 10.2196/22734 33684052 PMC8025920

[58] Li L, Erfani A, Wang Y, Cui Q, Anatomy into the battle of supporting or opposing reopening amid the COVID-19 pandemic on Twitter: A temporal and spatial analysis, *Plos One*, 2021;16 (7). doi: 10.1371/journal.pone.0254359 34255783 PMC8277023

