# Supplementary material for: A cross-country analysis of macroeconomic responses to COVID-19 pandemic using Twitter sentiments
Source: PLoS One. 2022 Aug 24;17(8):e0272208. doi: 10.1371/journal.pone.0272208 (PMC9401163; doi:10.1371/journal.pone.0272208)
Supplement: S1 Appendix — (PDF) [file pone.0272208.s001.pdf]

Appendix A

Gathering Twitter data

With the Twitter API academic researcher account up to ten million tweets can be pulled. Using a Twitter API academic researcher account, we retrieved geotagged tweets for Nigeria and South Africa from beginning of Jan 2017 until the end of February 2021. From 36682 number of tweets that were gathered, 20014 belonged to before and 16668 to during COVID-19 pandemic, i.e. since the beginning of March 2020 till now. The number of tweets with these keywords is highly correlated with the unemployment rate of South Africa. For Nigeria, the dataset contained 35501 tweets: 16050 of which are related to the COVID-19 pandemic and the rest to pre-COVID-19 times.

To make sure the chosen keywords are suitable for gathering a dataset from twitter for estimating the unemployment rates of Nigeria and South Africa, we checked the correlation between the volume of the gathered dataset and the unemployment rates. For years before COVID-19 pandemic, we used the seasonal census unemployment rates for correlation checking. However, during COVID-19 pandemic, since quarterly census measurements have not been able to capture the rapid unemployment rate changes and fluctuations caused by on and off rounds of lockdowns, we used the GMI for Correlation checking. Since it indicates how much people have moved around in different locations, it has a negative correlation with unemployment rate. We have averaged over five different indexes of GMI, i.e. retail and recreation, groceries and pharmacies, parks, transit stations, and workplaces, and excluded residential to find the right keywords for gathering a Twitter dataset for Nigeria and South Africa. Table A-1 shows the correlation of the number of tweets gathered with the unemployment rate and GMI for Nigeria and South Africa before and during COVID-19, and Table A-2 shows the correlation of the number of tweets gathered with these keywords with each other, before and during COVID-19 pandemic.

Table A-1. Correlation checking. Correlation of the number of tweets with GMI for Nigeria and South Africa

		employed	unemployed	retrench	lost * job	Total dataset
Unemployment Rate (before COVID-19)	Nigeria	0.78	0.8	-	0.59	0.77
GMI (during COVID-19)		-0.61	-0.6	-	-0.72	-0.74
Unemployment Rate (before COVID-19)	South Africa	0.9	0.88	0.83	-	0.92
GMI (during COVID-19)		-0.7	-0.71	-0.79	-	-0.83

Table A-2. Correlation checking. Correlation of the number of tweets with different keywords with each other for Nigeria and South Africa

			employed	unemployed	retrench	lost * job	Total dataset
		employed	1	0.99	-	0.74	0.99

Before COVID-19	Nigeria	unemployed		1	-	0.74	0.99
		lost * job			-	1	0.71
		total dataset			-		1
During COVID-19		employed	1	0.98	-	0.7	0.97
		unemployed		1	-	0.68	0.94
		lost * job			-	1	0.79
		total dataset			-		1
Before COVID-19	South Africa	employed	1	0.99	0.82	-	0.99
		unemployed		1	0.77	-	0.98
		retrench			1	-	0.89
		total dataset				-	1
During COVID-19		employed	1	0.98	0.5	-	0.95
		unemployed		1	0.47	-	0.93
		retrench			1	-	0.74
		total dataset				-	1

Fig A-1 shows a word cloud of our datasets for Nigeria and South Africa respectively, and Table A-3 shows the most common terms of our dataset and their frequencies.

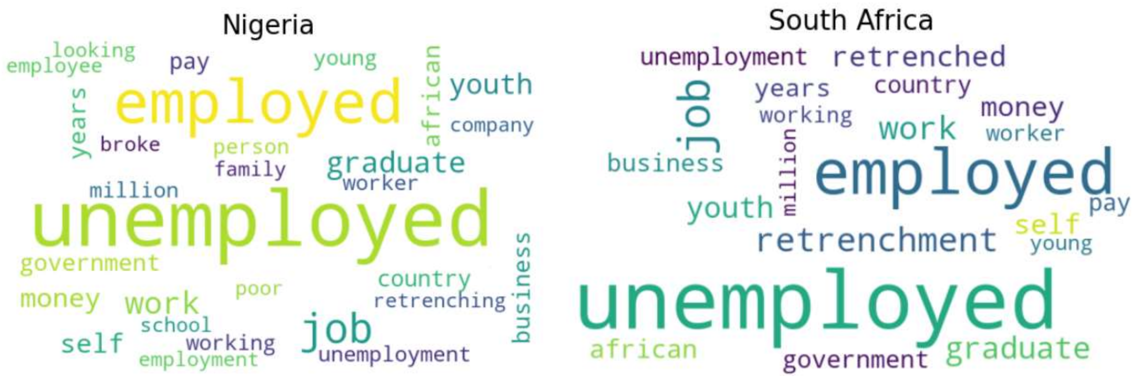

(A) Nigeria (B) South Africa

Fig A-1. Word Cloud. Word-Cloud for our datasets for (A) Nigeria and (B) South Africa

Table A-3. Term Frequency. number of repetitions for the most popular words of the datasets for Nigeria and South Africa

Nigeria		South Africa	
**Term**	**Frequency**	**Term**	**Frequency**
unemployed	30597	employed	30680

employed	18623	unemployed	18696
job	3677	work	3809
work	3583	job	3707
graduate	1733	retrenchment	2004
self	1863	graduate	1743
youth	1408	retrenched	1607
years	1243	self	1876
business	1204	pay	1501
country	1008	youth	1419
person	921	money	1346
government	827	African	1294
young	765	years	1276
million	752	business	1266
unemployment	725	country	1065
worker	719	working	911
employee	642	unemployment	876
looking	612	government	869
family	607	young	771
retrenching	596	worker	487
company	571		
African	47		

From Figs A-1(A) and A-1(B) and Table A-3 we can see that the popular words of the datasets for Nigeria and South Africa are very similar, both in the terms used and in their frequency.

Sentiment analysis

BERT is a model based on transformers that has been developed for Natural Language Processing (NLP), especially text classification. BERT has been trained for next sentence prediction and question answering in an unsupervised manner to learn the basic structure of the language. Because it is difficult to find labeled data for text classification and NLP in general, BERT has been trained and designed to have a high performance and accuracy in NLP with a smaller training dataset. BERT-large includes 24 layers of transformers with many parameters and thus, fine-tuning it is a very heavy process that needs a lot of resources. Therefore, most often BERT-base (which contains 12 layers of transformers) is used for fine-tuning. In this work, after gathering the tweets, we cleaned them by removing the URLs, punctuations, and stop-words. The tweets needed to be lemmatized to produce the word-clouds. To fine-tune BERT-base, we manually labeled 3600 tweets. About 42.21% of our data were neutral, 31.04% negative, and 26.75% positive. So, our dataset was reasonably balanced. We randomly choose 2000 of the labeled tweets for training, 800 for cross validation and 800 for testing. The tweets need to be tokenized by BERT tokenizer. The maximum number of tokens in both of our datasets were 63. We added one dropout layer with dropout popularity equal to 0.1 on top of BERT-base and a normal back-propagation layer with three outputs for the three sentiment classes (i.e. negative, neutral, and positive). Eventually, the final results went through a SoftMax layer to produce the probabilities of each sentiment polarity. We used Adaptive Moment Estimation with Weight Decay (AdamW) optimizer and cross entropy loss function to update the weights. The batch size was set to 32, and 20 number of epochs was enough to converge to the best accuracy. Fig A-2 shows the number of tweets predicted in each polarity class.

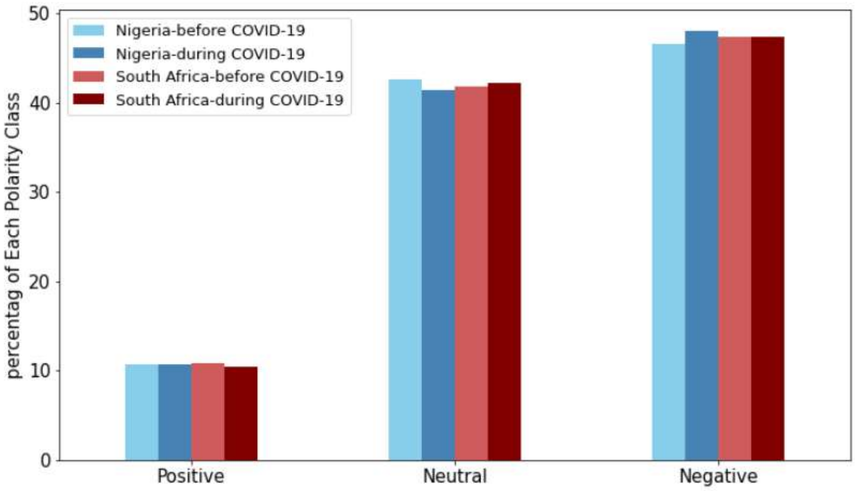

Fig A-2. Sentiment polarity. Number of tweets in each sentiment class for Nigeria and South Africa

According to Fig A-2 the polarity of the sentiments for both countries are very close to each other. Since the dataset is on unemployment, most of the tweets are negative, then neutral, and very few of them are positive. The tweets from Nigeria are slightly more negative than the tweets from South Africa. Tweets from Nigeria have become more negative and less neutral during COVID-19 pandemic compared to before that. Moreover, the tweets from South Africa have become more neutral and less positive during COVID-19 pandemic compared to before that. In general, the polarity of the sentiments has slightly decreased during COVID-19 pandemic.

We found a strong negative correlation between the unemployment rate and the sentiment scores in South Africa and Nigeria. After placing a SoftMax layer on top of the sentiment analysis model, the probability of the sentiment belonging to each class is obtained. Using these probabilities, the sentiment scores are calculated. Furthermore, Fig A-3 shows the unemployment rate and the sentiment scores of our model before and during COVID-19 pandemic for Nigeria, and Fig A-4 shows that for South Africa.

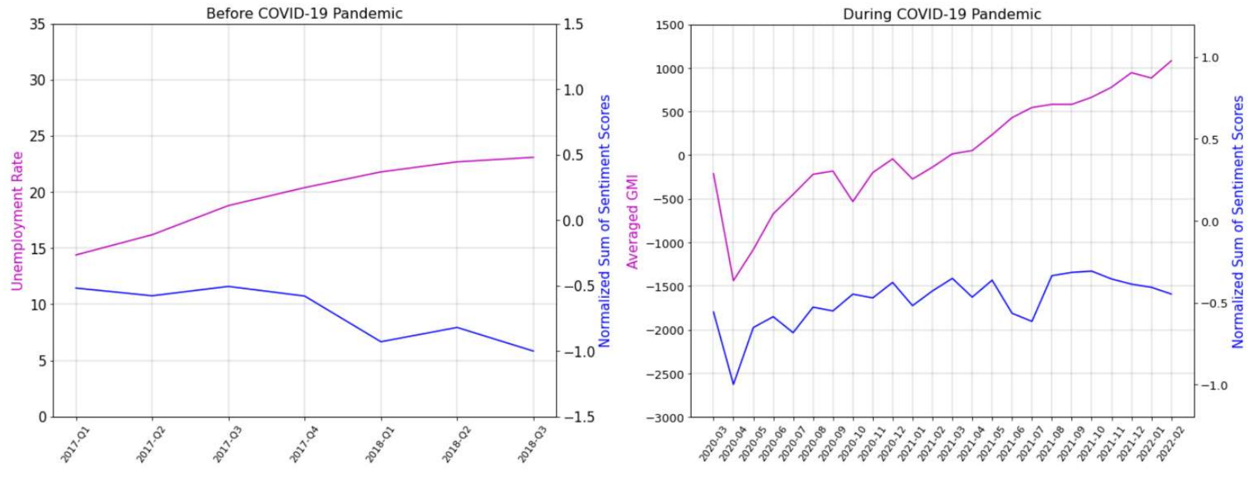

(A) **(B)**

Fig A-3. Sentiment scores. Normalized sum of sentiment scores and unemployment rate (A) before and (B) during COVID-19 for Nigeria

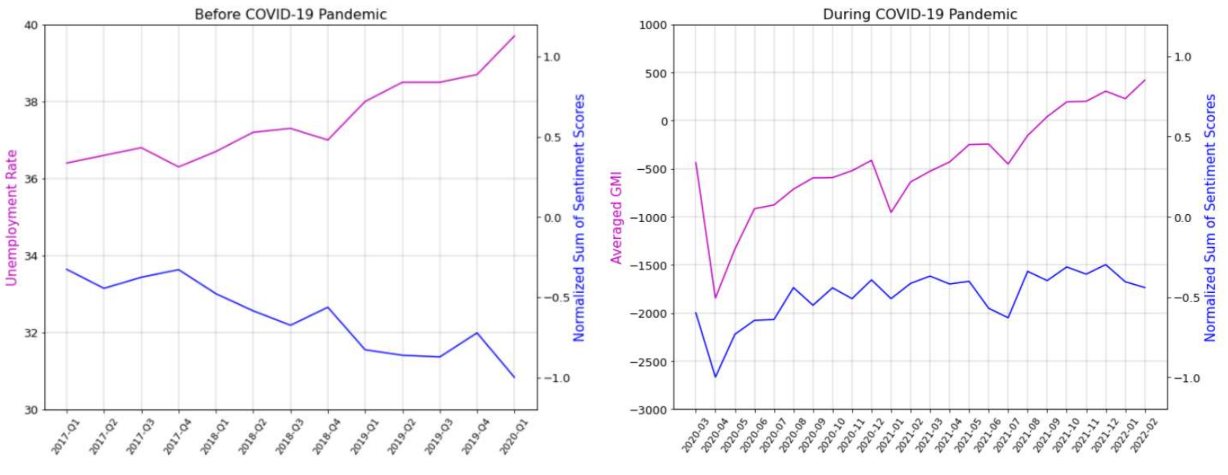

(A) **(B)**

Fig A-4. Sentiment scores. Normalized sum of sentiment scores and unemployment rate (A) before and (B) during COVID-19 for South Africa

As can be seen in figures above, sentiment scores have a negative value and are negatively correlated with the unemployment rate. This is expected as the dataset is on unemployment rate. Moreover, this shows that as the unemployment rate increases people become more dissatisfied and the sentiment scores decrease, in contrast, when the unemployment rate decreases, sentiment scores increase. Through four different machine learning algorithms and their average, both the volume and the sentiment of the tweets were used to predict the unemployment rate of Nigeria and South Africa. Figure A-5 shows our algorithm for predicting the unemployment rate of Nigeria and South.

Algorithm1: Predicting monthly unemployment rates using census measures.

Input: census[] : a set containing the unemployment rates. The census measurement is available for the known months and 0 is placed for the rest.

Output: unemployment_rate[]: predicted monthly unemployment rates.

```
model = ϕ
known_months = [ ]
for each item in census:
  if item == 0 and model == ϕ:
    unemployment_rate [item] = 0
  else if item ≠ 0 and model == ϕ:
    unemployment_rate [item] = 0
    known_months.add (item)
    model.train (known_months)
  else if item ==0 and model ≠ ϕ:
    unemployment_rate [item] = model.forecast (item)
  else if item ≠ 0 and model ≠ ϕ:
    unemployment_rate [item] = model.forecast (item)
    known_months.add (item)
    model.train (known_months)
return unemployment_rate
```

Fig A-5. Prediction algorithm. Algorithm for predicting unemployment rates
